# Ice in Cholera Infantum

**Published:** 1841-11

**Authors:** W. S. Lawrie

**Affiliations:** New Concord, Ky.


**Art. V.**—*Ice in Cholera Infantum.* By Dr. W. S. Lawrie, of New Concord, Ky.

In the last number of the Journal I observe a recommendation of *ice* in cholera infantum. It is the first and only instance in which I have seen this article publicly recommended in this truly formidable disease. My attention was directed to its employment while under the tuition of Dr. Jackson, of Jackson, Tenn. In my own practice, it has been uniformly exhibited, when practicable to obtain it; and though my professional experience is limited, in all obstinate cases of this disease, I have not failed to prescribe it with most decided benefit. In the article in the Journal it is stated, that the most disagreeable retching, always troublesome, was almost instantly relieved by administering ice. From personal experience, I am able fully to confirm this affirmation; and I have frequently found, that while almost every thing else exhibited, and particularly in liquid form, was at once ejected, the ice, freely given, seemed to possess the power of allaying the excessive irritability of the stomach, and of alleviating the sufferings of the patient.

Another article from which, in conjunction with ice, much benefit may be derived in cholera infantum, is good port wine. In addition to what ice the child will eat, I am in the habit of using the wine made into sangaree, *freely iced*, giving such quantity as it may be induced to take.

*September*, 1841.

